# Expansion of Schizophrenia Gene Network Knowledge Using Machine Learning Selected Signals From Dorsolateral Prefrontal Cortex and Amygdala RNA-seq Data

**DOI:** 10.3389/fpsyt.2022.797329

**Published:** 2022-03-21

**Authors:** Yichuan Liu, Hui-Qi Qu, Xiao Chang, Lifeng Tian, Joseph Glessner, Patrick A. M. Sleiman, Hakon Hakonarson

**Affiliations:** ^1^Center for Applied Genomics, Children's Hospital of Philadelphia, Philadelphia, PA, United States; ^2^Department of Pediatrics, The Perelman School of Medicine, University of Pennsylvania, Philadelphia, PA, United States; ^3^Division of Human Genetics, Children's Hospital of Philadelphia, Philadelphia, PA, United States; ^4^Division of Pulmonary Medicine, Children's Hospital of Philadelphia, Philadelphia, PA, United States; ^5^Faculty of Medicine, University of Iceland, Reykjavik, Iceland

**Keywords:** schizophrenia, machine learning, biological network, amygdala, dorsolateral prefrontal cortex (DLFPC)

## Abstract

It is widely accepted, given the complex nature of schizophrenia (SCZ) gene networks, that a few or a small number of genes are unlikely to represent the underlying functional pathways responsible for SCZ pathogenesis. Several studies from large cohorts have been performed to search for key SCZ network genes using different analytical approaches, such as differential expression tests, genome-wide association study (GWAS), copy number variations, and differential methylations, or from the analysis of mutations residing in the coding regions of the genome. However, only a small portion (<10%) of candidate genes identified in these studies were considered SCZ disease-associated genes in SCZ pathways. RNA sequencing (RNA-seq) has been a powerful method to detect functional signals. In this study, we used RNA-seq data from the dorsolateral prefrontal cortex (DLPFC) from 254 individuals and RNA-seq data from the amygdala region from 46 individuals. Analysis was performed using machine learning methods, including random forest and factor analysis, to prioritize the numbers of genes from previous SCZ studies. For genes most differentially expressed between SCZ and healthy controls, 18 were added to known SCZ-associated pathways. These include three genes (*GNB2, ITPR1*, and *PLCB2*) for the glutamatergic synapse pathway, six genes (*P2RX6, EDNRB, GHR, GRID2, TSPO*, and *S1PR1*) for neuroactive ligand–receptor interaction, eight genes (*CAMK2G, MAP2K1, RAF1, PDE3A, RRAS2, VAV1, ATP1B2*, and *GLI3*) for the cAMP signaling pathway, and four genes (*GNB2, CAMK2G, ITPR1*, and *PLCB2*) for the dopaminergic synapse pathway. Besides the previously established pathways, 103 additional gene interactions were expanded to SCZ-associated networks, which were shared among both the DLPFC and amygdala regions. The novel knowledge of molecular targets gained from this study brings opportunities for a more complete picture of the SCZ pathogenesis. A noticeable fact is that hub genes, in the expanded networks, are not necessary differentially expressed or containing hotspots from GWAS studies, indicating that individual methods, such as differential expression tests, are not enough to identify the underlying SCZ pathways and that more integrative analysis is required to unfold the pathobiology of SCZ.

## Introduction

Schizophrenia (SCZ) is a chronic and severely disabling neurodevelopmental disorder that affects people of all races and background worldwide. The redundancy of the gene networks underlying SCZ indicates that many gene combinations have the potential to result in a brain dysfunction that can manifest as SCZ or a related neurodevelopmental disorder (1, 2). Next-generation sequencing enables one to measure the transcriptome gene expression through RNA-seq; however, recent studies from large cohorts show that differentially expressed genes alone are not the final solution to understand the molecular underpinnings underlying SCZ networks, as a number of biological processes, such as methylation, eQTLs. and copy number variations (CNVs) in the DNA sequence, are among the biological processes that contribute to the biological pathways of SCZ (3–8). Almost 6,000 genes with supportive evidence from these studies were identified, but only a small portion (<10%) could be labeled as SCZ-associated genes based on information from disease databases, and even a smaller portion of these genes was found to be functionally relevant to SCZ-related biological pathways.

In this study, we selected the most informative genes that demonstrated differential expression/variation between SCZ and healthy controls based on previous studies; we then added them into known SCZ-associated pathways, and we subsequently reworked the SCZ-associated pathways using an experimental gene interaction database. In other words, we applied machine learning methods, including random forest and factor analysis, on transcriptome RNA sequencing (RNA-seq) data generated from 254 human dorsolateral prefrontal cortex (DLPFC) samples and 46 human post-mortem amygdala samples, which covered all genes previously linked to SCZ with supportive evidence, to identify the driving biological signals representing SCZ in brain tissues. We found that two different brain loci (DLPFC and amygdala) show certain levels of similarities, and only small portions of hub genes in expanded networks are differentially expressed at the RNA level. These results suggest that SCZ gene interactions are likely functionally impacting multiple locations of the brain and that platforms capturing multiple different domains of molecular data need to be integrated to reveal the entire picture of the gene networks underlying SCZ.

## Methods and Materials

### Selections for SCZ Genes With Supportive Evidence From Previous Studies

Differentially expressed SCZ genes were selected from the previous study on post-mortem DLPFC tissues from 258 SCZ cases and 279 controls with European, African American, Hispanic, and East Asian ancestries (9). Genome-wide association study (GWAS) gene targets were collected through the Psychiatric Genomics Consortium GWAS (10) (36,989 cases and 113,075 controls) and the CLOZUK GWAS (7) (11,260 cases and 24,542 controls). Differential methylation genes were collected based on multiple literatures (3, 4, 11). Genes impacted by copy number variations (CNVs) were collected based on a comparative study of 21,094 cases and 20,227 controls (6). Genes with eQTL hits were collected based on multiple recently published literatures (5, 8, 12). The genes identified in linkage studies were collected based on the meta-analysis of 32 genome-wide linkage studies of schizophrenia (13). The genomic variation of SCZ genes was extracted from multiple exome sequencing studies (14–16). The gene expression levels in brain tissues were obtained from the GeneCard database. All the extracted genes were merged to form the “gene pool” for selection processes. In total, there are 460 GWAS genes, 223 genes identified in previous linkage studies, 392 genes containing SCZ-associated CNVs, 3,540 genes with at least one SCZ-correlated exome mutation, 1,890 genes with differential methylation, and 683 genes differentially expressed in SCZ case/control studies.

### Identification of SCZ-Associated Genes Based on Disease Database

The selected SCZ genes with at least one supportive evidence were considered as the background or the “gene pool” for analysis. Genes from the pool that were identified in a disease database, including DisGeNET (17), GLAD4U (18), and Online Mendelian Inheritance in Man, were categorized as “SCZ-associated genes” or “gene set A,” while the rests in the gene pool were categorized as “gene set B.” The gene enrichment analysis was performed by DAVID bioinformatics platform (19) and WebGestalt (20).

### RNA-Seq Data for Dorsolateral Prefrontal Cortex and Amygdala

The RNA-seq data of DLPFC samples were obtained from the CommonMind consortium FTP sites directly. To eliminate the confounding effects of different populations, we only selected SCZ patients and controls who are of European ancestry (EA). A total of 254 RNA-seq BAM files were obtained, including 120 SCZ patients and 134 healthy controls. The samples with a minimum of 50 million mapped reads and <5% rRNA-aligned reads were retained for downstream analysis. In total, 46 post-mortem amygdala tissues, including 22 SCZ patients and 24 healthy controls, were obtained. Like the DLPFC samples, all individuals for amygdala tissues are EA. More details of the samples and sequencing procedures could be found in the previous publication (21).

### RNA Expression Matrix

The genomic template used for coding the genes' expressions is hg19 refSeq, and the long non-coding RNA template is GENCODE version 19 (22). The expression matrix was generated based on Cuffnorm functions in Cufflink package version 2.2.1 (23), and the SCZ and controls groups are normalized. Additional quality control (QC) of expression data was performed in accordance with Sheng et al. (24). To eliminate potential noise signals, genes with expression of FPKM values <1 and genes with collinearity over 80% were removed. Further QCs were adopted in the following machine learning algorithms.

### Gene Selection Using Machine Learning Algorithms

Machine learning algorithms, including random forest and factor analysis, were applied to select and reduce the informative gene features between SCZ cases and controls for DLPFC and amygdala, respectively. Random forest is one of the most widely used algorithms for feature selection, which computes the relative importance or contribution of each gene feature in the prediction and then scales the relevance down so that the sum of all scores is 1. All the genes with zero relative importance were removed. The modeling codes are based on the Scikit-learn package in Python language (25).

Factor analysis was applied to the entire sample set for further clustering gene features. Factor analysis is a statistical method used to describe the variability among observed, correlated variables in terms of a potentially lower number of unobserved variables called factors, and the methods have been proven to be a good interpreter for gene networks and pathways. The Python-based factor_analyzer package was used in the analysis.

### Expanding SCZ-Associated Pathways and Networks

The Kyoto Encyclopedia of Genes and Genomes database (data release version 2020/04) was applied as a pathway reference for SCZ-associated pathway analysis (26), and the gene pool described in the previous section was used as background for enrichment analysis. Gene set A, which is composed of SCZ-associated genes identified in a disease database, was examined in an enrichment analysis, and the corresponding pathways were considered as SCZ-associated pathways. Gene set B that remained in the selection processes from factors that represent top variances went through an enrichment analysis again with set A, and the genes in set B were considered as candidate genes of SCZ-associated pathways if they were assigned to the same pathways enriched with set A genes. In other words, the newly added candidate genes of SCZ-associated pathways must be in the corresponding pathway already but have not been identified in disease databases previously.

The SCZ-associated pathways remained significant after false discovery rate (FDR) adjustments were further expanded into networks that do not require candidate genes from set B identified in the pathways. Gene interaction databases, such as BioGrid (27), were applied in the expansion procedures, and the visualizations were done by CytoScape (28). Hub genes were extracted from the developed networks based on the number of interactions (degree of connectivity).

## Results

### Candidate Genes in SCZ-Associated Pathways Based on DLPFC

There are 5,948 genes with at least one supportive evidence of SCZ involvement from previous SCZ studies, and these genes served as the gene pool for our study. Of those, 534 SCZ-associated genes (~9%) (set A) were identified through the SCZ disease database, which left 5,414 genes out (set B). The enrichment analysis for set A using the pool as background revealed five SCZ-associated pathways, including 35 genes found in dopaminergic synapse networks (FDR = 2.7 × 10^−13^), 49 genes in neuroactive ligand–receptor interaction networks (1.6 × 10^−12^), 26 genes in glutamatergic synapse pathways (1.9 × 10^−10^), 34 genes in cAMP signaling pathways (6.4 × 10^−8^), and 24 genes in serotonergic synapse pathways (7.6 × 10^−7^).

**Figure 1:**
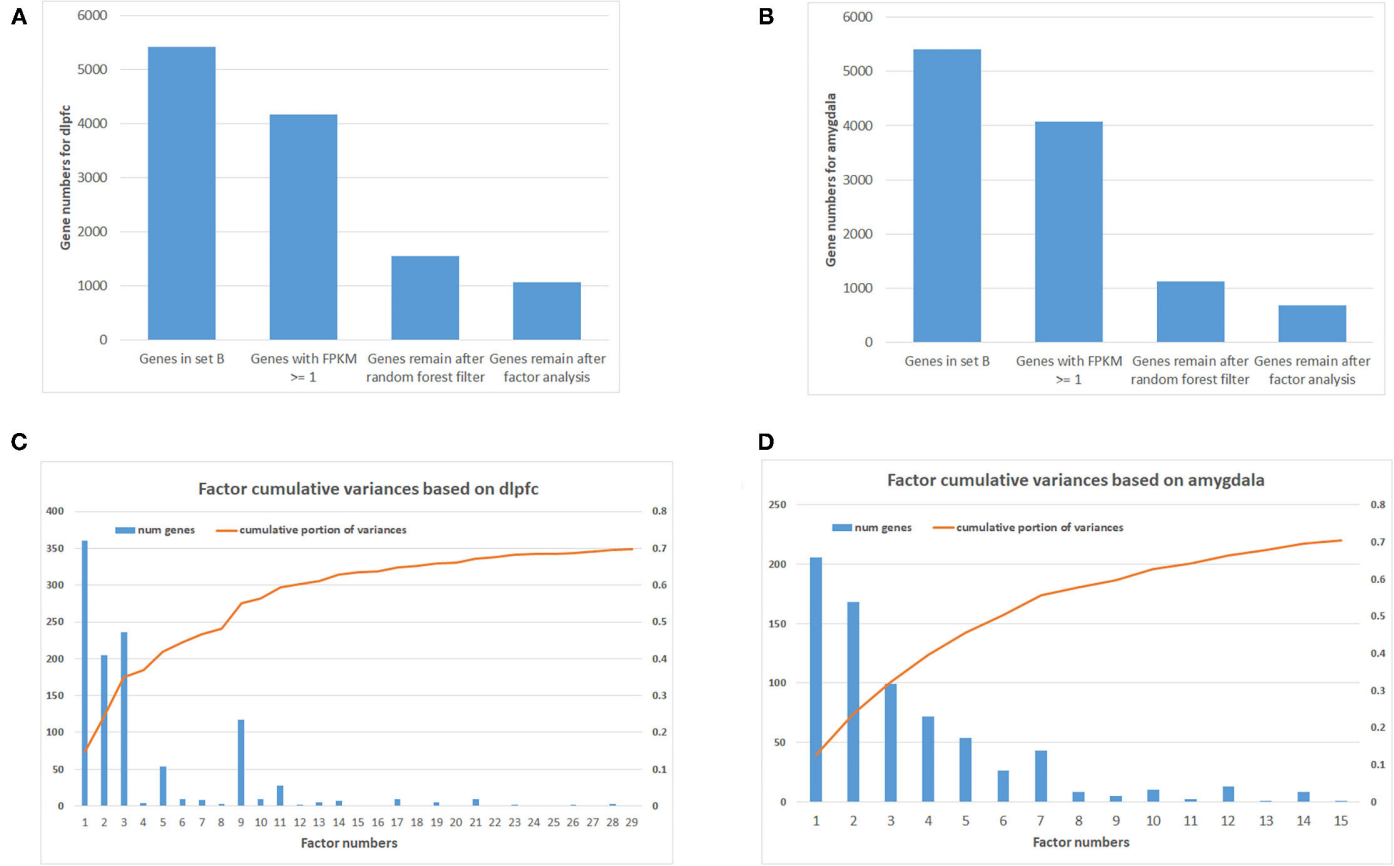
Number of genes after filtering and factor analysis cumulative curve. **(A)** Number of genes after multiple filtering methods for dorsolateral prefrontal cortex (DLPFC). **(B)** Number of genes after multiple filtering methods for amygdala. **(C)** Factor analysis cumulative curve and number of remaining genes for DLPFC. **(D)** Factor analysis cumulative curve and number of remaining genes for amygdala.

Using machine learning methods, including random forest followed by factor analysis, on 254 DLPFC samples (120 SCZ vs. 134 controls) reduced the number of informative genes in set B from 5,414 to 1,068 genes (top 29 factors represent 70% variances between SCZ and controls; Figures 1A,C). Each factor was combined with set A, and enrichment analysis was performed again to assess whether previous SCZ-associated pathways remained associated, and if so, the genes (i.e., factors), which were in the pathway but not in set A, were considered as candidate SCZ-associated genes. This resulted in 18 SCZ-associated genes being identified that matched with the previous five SCZ-associated pathways (Table 1), including *GNB2, CAMK2G*, P*2RX6, MAP2K1*, and *RAF1* from factor 1, *CYP2D6* and *ITPR1* from factor 2*, EDNRB, GHR, GRID2, PDE3A*, and *RRAS2* from factor 3, *S1PR1, ATP1B2*, and *GLI3* from factor 5, and *PLCB2, TSPO*, and *VAV1* from factor 9. Notably, some candidate genes showed up in multiple SCZ-associated pathways, suggesting that they may confer higher impacts than other genes—for example, *GNB2* is involved as a modulator/transducer of various transmembrane signaling systems and activator of kainate receptors upon glutamate binding (29). More specifically in the glutamatergic synapse pathway, *GNB2* controls exocytosis in the presynaptic terminal by inhibition of glutamate releases and interaction with the GRM family genes *GRM2, GRM3, GRM4, GRM7*, and *GRM8*. In the dopaminergic synapse pathway, *GNB2* takes signals from the DRD family and delivery to the PLC gene family to regulate neural excitability. Previous studies show that *GNB2* and its coding proteins are highly expressed in brain tissue, and *de novo* mutations in *GNB2* show effects on synaptic proteins and genes involved in schizophrenia and other neuropsychiatric diseases (14). The *ITPR1* gene provides instructions for channels that control the flow of calcium ions. The *ITPR1* channel delivers Ca^++^ from calcium signaling pathways to protein kinase C, regulating synaptic plasticity *via* IP3 signals from *PLCB* in dopaminergic synapse pathways. *ITPR1* interacts directly with *GRM1* and *GRM5* genes in the glutamatergic synapse pathway. The CNV duplications in *ITPR1* were shown to be associated with attention deficit/hyperactivity disorder (ADHD) and autism spectrum disorder (ASD) (30), and CNVs in genes at the chr3p26 locus, including but not limited to *CNTN4*, have been associated with ADHD and ASD and shown to impair glutamatergic signaling (31, 32).

**Table 1:** Results from an integrative analysis uncovering 18 schizophrenia-associated candidate genes and corresponding pathways.

**Genes**	**Factor**	**Dopaminergic synapse pathway**	**Neuroactive ligand–receptor interaction**	**Glutamatergic synapse pathway**	**cAMP signaling pathway**	**Serotonergic synapse**
GNB2	1	Y	-	Y	-	Y
CAMK2G	1	Y	-	-	Y	-
P2RX6	1	-	Y	-	-	-
MAP2K1	1	-	-	-	Y	Y
RAF1	1	-	-	-	Y	Y
CYP2D6	2	-	-	-	-	Y
ITPR1	2	Y	-	Y	-	-
EDNRB	3	-	Y	-	-	-
GHR	3	-	Y	-	-	-
GRID2	3	-	Y	-	-	-
PDE3A	3	-	-	-	Y	-
RRAS2	3	-	-	-	Y	-
S1PR1	5	-	Y	-	-	-
ATP1B2	5	-	-	-	Y	-
GLI3	5	-	-	-	Y	-
PLCB2	9	Y	-	Y	-	Y
TSPO	9	-	Y	-	-	-
VAV1	9	-	-	-	Y	-

### SCZ-Associated Pathway Expansion to Networks Using Gene Interaction Database

Besides selecting candidate genes within identified SCZ-associated pathways, the pathways were further expanded to networks based on gene interaction database analysis. In other words, genes in set A that were identified in SCZ-associated pathways, including 35 genes in dopaminergic synapse pathways, 49 genes in neuroactive ligand–receptor interaction networks, 26 genes in glutamatergic synapse pathways, 34 genes in cAMP signaling pathways, and 24 genes in serotonergic synapse networks, were connected to factor analysis-identified genes through gene interaction database analysis to form potential SCZ networks. Using 254 DLPFC samples and interactions identified in BioGrid, as described in the method section, 265 direct interactions were built between set A genes in dopaminergic synapse pathways and set B genes selected by factor analysis, including 85 interactions for neuroactive ligand–receptor interaction networks, 71 interactions for glutamatergic synapse pathways, 239 interactions for cAMP signaling pathways, and 78 interactions for serotonergic synapse pathways.

**Figure 2:**
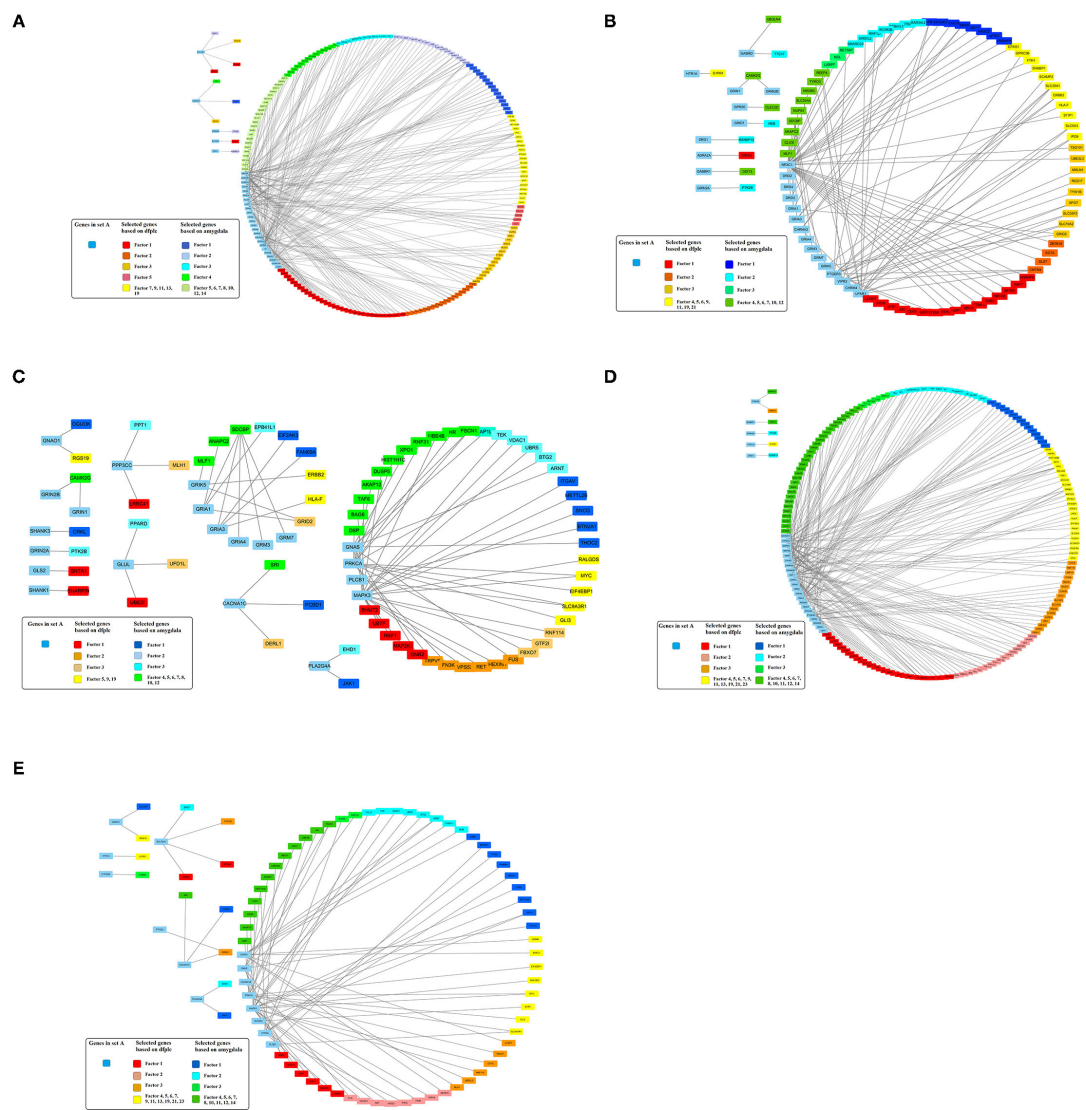
Expanded schizophrenia-associated pathways using machine learning selected genes in dorsolateral prefrontal cortex union amygdala based on gene interaction database. **(A)** Dopaminergic synapse pathway. **(B)** Neuroactive ligand–receptor interaction. **(C)** Genes in glutamatergic synapse pathway. **(D)** cAMP signaling pathway. **(E)** Serotonergic synapse pathway.

In addition, 46 amygdala brain samples were also analyzed independently besides DLPFC to check the specificity and consistency of gene networks between the two major functional regions of the brain in SCZ. To control confounding effects from population differences, we limited the ethnicity to European ancestors for both cases and controls. Similar patterns were seen in amygdala (Figures 1B,D), with 1,119 genes remaining in the factor analysis (677 genes in the top 15 factors represent 70% variances between SCZ cases *vs*. controls). Of those, 464 (41.7%) genes remained in the factor analysis for amygdala and were also identified in DLPFC, including 185 (27.3%) genes in the top factors. The results suggest that some SCZ networks are consistent among different functional loci of the brain. The differences could be due to (and explain) functional alterations within these two brain regions, whereas they may also be due to technical reasons, such as sample size differences, batch effects, *etc*. In the network expansion processes, 177 direct interactions were built between set A genes in dopaminergic synapse pathways and the set B genes that remained in the factor analysis for amygdala, including 62 interactions for neuroactive ligand–receptor interaction networks, 55 interactions for glutamatergic synapse pathways, 141 interactions for cAMP signaling pathways, and 54 interactions for serotonergic synapse pathways. A total of 103 interactions are found in both DLPFC and amygdala (Table 2). The union of networks for both loci provides a more complete picture of SCZ-associated networks upon expansions (Figures 2A–E).

**Table 2:** Interactions in both dorsolateral prefrontal cortex (DLPFC) and amygdala from expanding schizophrenia-associated networks.

**Set A genes**	**Set B genes remaining in top factors**	**Factor number (DLPFC)**	**Factor number (amygdala)**	**Pathway**
CACNA1C	PCBD1	1	1	
CASP3	DBNL	1	1	
GNAS	FSCN1	1	4	
GNAS	XPO1	3	5	
HTR3A	FITM2	1	1	
HTR3A	HIST1H1C	9	6	
MAPK3	TEK	3	2	Serotonergic synapse pathway
MAPK3	DUSP5	19	6	
PLA2G4A	JAK1	3	1	
PRKCA	FSCN1	1	4	
PRKCA	HIST1H1C	9	6	
PRKCA	AKAP12	3	7	
SLC18A1	EMC7	3	2	
CHRNA3	TMEM219	1	1	
GABBR1	DDIT3	1	6	
GRIN1	CAMK2G	1	4	
GRIN2A	PTK2B	2	2	
GRIN2B	CAMK2G	1	4	Neuroactive ligand–receptor interaction
LPAR1	FITM2	1	1	
NR3C1	SMARCC2	2	2	
PTGER3	RETSAT	1	3	
VIPR2	FITM2	1	1	
CACNA1C	PCBD1	1	1	
GNAS	FSCN1	1	4	
GNAS	XPO1	3	5	
GRIN1	CAMK2G	1	4	
GRIN2A	PTK2B	2	2	
GRIN2B	CAMK2G	1	4	
MAPK3	TEK	3	2	Glutamatergic synapse pathway
MAPK3	DUSP5	19	6	
PLA2G4A	JAK1	3	1	
PRKCA	FSCN1	1	4	
PRKCA	HIST1H1C	9	6	
PRKCA	AKAP12	3	7	
SHANK3	CRKL	2	1	
AKT1	SMARCC2	2	2	
AKT1	FAM110C	3	2	
AKT1	TEK	3	2	
AKT1	DCTN1	1	4	
AKT1	TCOF1	2	4	
ARRB2	SF3B1	3	2	
ARRB2	SMARCC2	2	2	
ARRB2	RPLP0	9	2	
ARRB2	RPL22	3	3	
ARRB2	TCOF1	2	4	
ARRB2	XPO1	3	5	
ARRB2	HIST1H1C	9	6	
ARRB2	SF3B2	1	10	
CACNA1C	PCBD1	1	1	
CALM1	SF3B1	3	2	
CALM1	RPL22	3	3	
CALM1	CAMK2G	1	4	
CAMK2A	DBNL	1	1	Dopaminergic synapse pathway
CAMK2A	ARL3	3	4	
CAMK2A	CAMK2G	1	4	
CAMK2A	DCTN1	1	4	
GNAS	FSCN1	1	4	
GNAS	XPO1	3	5	
GRIN2A	PTK2B	2	2	
GRIN2B	CAMK2G	1	4	
GSK3A	RBM8A	1	3	
GSK3B	C14orf1	3	1	
GSK3B	SF3B1	3	2	
GSK3B	TLE1	2	4	
GSK3B	RNF220	1	4	
GSK3B	XPO1	3	5	
PPP2R2B	PPP4C	1	1	
PRKCA	FSCN1	1	4	
PRKCA	HIST1H1C	9	6	
PRKCA	AKAP12	3	7	
SLC18A1	EMC7	3	2	
AKT1	SMARCC2	2	2	
AKT1	FAM110C	3	2	
AKT1	TEK	3	2	
AKT1	DCTN1	1	4	
AKT1	TCOF1	2	4	
CACNA1C	PCBD1	1	1	
CALM1	SF3B1	3	2	
CALM1	RPL22	3	3	
CALM1	CAMK2G	1	4	
CAMK2A	DBNL	1	1	
CAMK2A	ARL3	3	4	
CAMK2A	CAMK2G	1	4	
CAMK2A	DCTN1	1	4	
GABBR1	DDIT3	1	6	
GNAS	FSCN1	1	4	
GNAS	XPO1	3	5	cAMP signaling pathway
GRIN1	CAMK2G	1	4	
GRIN2A	PTK2B	2	2	
GRIN2B	CAMK2G	1	4	
MAPK3	TEK	3	2	
MAPK3	DUSP5	19	6	
PDE4B	XPO1	3	5	
PDE4D	AKAP12	3	7	
PTGER3	RETSAT	1	3	
RELA	PPP4C	1	1	
RELA	SETD6	3	1	
RELA	MKRN2	3	1	
RELA	MACROD1	1	1	
RELA	AATF	3	2	
RELA	TLE1	2	4	
RELA	XPO1	3	5	
VIPR2	FITM2	1	1	

## Discussion

The DLPFC dysfunction is one of the most important differentiators in the pathogenesis of SCZ (33). DLPFC is an area in the prefrontal cortex that connects the thalamus and the hippocampus. DLPFC is important in many brain functions, such as working memory, planning, and reasoning. Previous studies suggest an association between decreased DLPFC activity and disabling disorganization symptoms and memory deficits in individuals with SCZ (33, 34). The consequences of amygdala dysfunction have also been highlighted in SCZ (35). Anatomically, amygdala consists of two almond-shaped groups of nuclei located deep and medially within the temporal lobes of the human brain. Functionally, the amygdala plays a primary role in the processing of memory, decision-making, and emotional reactions, and previous studies suggest that dysregulation of the amygdala region contributes to the pathogenesis of SCZ (2). As a result, combining the networks built from both loci would provide an expanded view and potentially new knowledge of SCZ gene networks.

Multiple SCZ studies have been performed in large cohorts to explore potential key regulators of SCZ networks, including differential tests for gene expression and methylation, GWASs, CNVs, eQTLs, and mutation studies based on exome sequencing data. Around 6,000 genes have been identified with supportive evidence of their contribution to SCZ, consistent with the assumption that SCZ is a highly complex biological disorder involving the combined effects of many genes, each conferring a small increase in susceptibility to the illness. On the other hand, the selection for informative signals from a large number of genes (28% of the entire human coding genes) is challenging and becomes the main obstacle to pinpoint the essential key drivers of the SCZ network regulators. So far, only small portions (~9%) of genes with supportive evidence for SCZ involvement have been mapped to SCZ-associated pathways. Machine learning methods have been proven to be effective in reducing the feature vectors while capturing essential data differences in studies of many fields, including genetic expression studies (36). Therefore, in this study, we applied random forests and factor analysis involving DLPFC and amygdala RNA-seq expression data to select the most informative gene signals in SCZ by merging results from the two brain regions that have been implicated in SCZ, taking an experimentally based gene interaction database approach.

To facilitate research on psychiatric diseases, the PsychENCODE project produces a public resource of genomic data using tissue- and cell type-specific samples (37). As a resource of PsychENCODE, the CommonMind Consortium provides transcriptomic and epigenomic data for SCZ and bipolar disorder (38). Based on 534 SCZ-associated genes from disease databases, we identified five well-replicated and significantly enriched SCZ pathways that are critical in the neurodevelopment processes underlying SCZ, including dopaminergic synapse pathways, neuroactive ligand–receptor interaction networks, glutamatergic synapse pathways, cAMP signaling pathways, and serotonergic synapse pathways. This approach uncovered 18 genes from the factor analysis which belong to these same pathways and were incorporated as SCZ-associated gene candidates. Consequently, these pathways have been expanded based on gene interaction database information to broaden the SCZ knowledgebase. As a result, 294 interactions based on DLPFC and 216 interactions based on amygdala, including 103 interactions identified in both loci, have been incorporated to inform SCZ pathogenesis. These data underline the interactive gene networks that exist between DLPFC and amygdala and emphasize the different functions of these loci that may be uniquely implicated in SCZ pathogenesis. The new knowledge gained from this study could bring opportunities to conquer SCZ by targeting these new molecular targets using innovative precision-based strategies.

**Figure 3:**
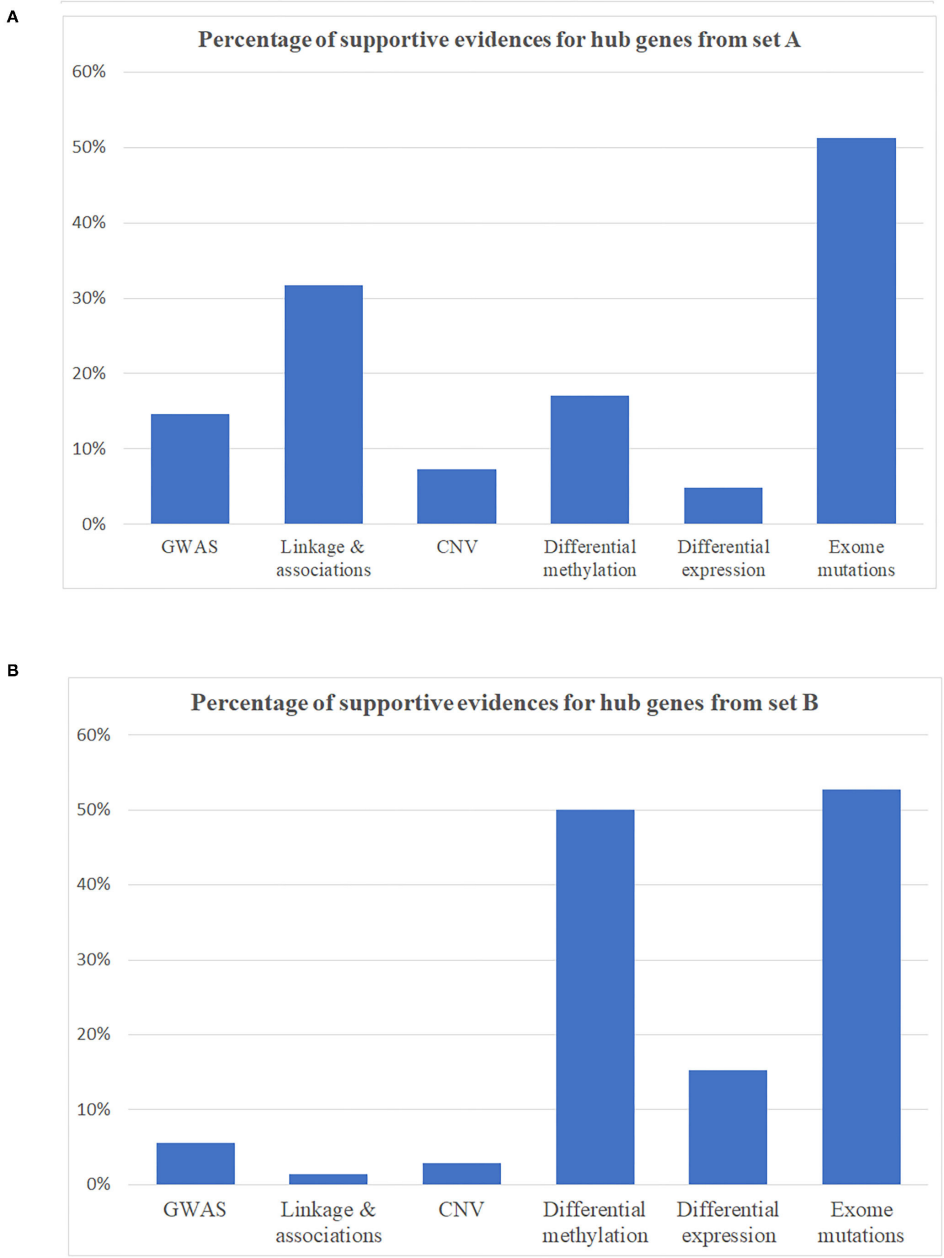
Percentage of supportive evidence for hub genes in expanding networks. **(A)** Hub genes from disease identified as schizophrenia-associated genes (gene set A). **(B)** Hub genes from gene set B.

Hub genes, which have more than one edge in the expanded networks, usually have more impact on the entire networks due to high connectivity within the networks. As a result, hub genes in the expanded networks were extracted and explored in further detail (Table 3). Notably, the weaker the support, such as single analysis support only, the more it is inversely correlated with the number of hub genes (Figures 3A,B)—for example, among hub genes identified within the 534 identified SCZ-associated gene set (set A), only two of them (4.8%) were captured by differential expression alone and six of them (14.6%) were uncovered by GWAS. For the hub genes from set B, the portion is 15.2% from differential expression tests and 5.6% from GWAS study. These results suggest that a more integrative analysis is warranted to enrich the number and role of genes mapping to SCZ gene networks, and studies focused on defined aspects may have limitations to reveal the broader picture of SCZ pathogenesis. The direction of effects was not emphasized in this study mainly because conflicts of the evidence when integrating databases from independent studies are inevitable and addressing these conflictions may cause the negligence of interesting genes in this study—for example, the hub gene*FSCN1* shown in Table 3, which contains a potential damaging *de novo* missense mutation p.E162D, is hypomethylated in schizophrenia but has a low gene expression compared to controls. On the other hand, the hub gene *DERL1* is hypomethylated in schizophrenia but with a higher gene expression. Another limitation for this study is that candidate genes from the previous genetic studies were mainly selected based on physical proximity in the human genome and might not represent a functional link. More extensive gene and gene set analysis in those genetic regions, e.g., using Multi-marker Analysis of GenoMic Annotation (39), is warranted, especially for the genes as potential novel therapeutic targets.

**Table 3:** Hub genes from known SCZ-associated genes (gene set A) and from gene set B with supportive evidence from at least two pathways.

**GeneID**	**Pathway**	**GWAS**	**Linkage association studies**	**CNV**	**Differential methylations**	**Differential expression**	**Exome mutations**	**Brain expression**
**Hub genes from gene set A**								
CACNA1C	mGluR, dopamine synapse, cAMP, serotonergic synapse	1	0	0	1	0	1	High
GNAS	mGluR, dopamine synapse, cAMP, serotonergic synapse	0	0	0	1	0	1	High
GRIA1	mGluR, dopamine synapse, cAMP, neuroactive ligand–receptor interaction	0	1	0	0	0	0	High
GRIA3	mGluR, dopamine synapse, cAMP, neuroactive ligand–receptor interaction	0	0	0	0	0	1	High
DRD2	Dopamine synapse, neuroactive ligand–receptor interaction, cAMP	1	1	0	0	0	0	Low
GNAO1	mGluR, dopamine synapse, serotonergic synapse	0	0	0	0	0	1	High
MAPK3	mGluR, cAMP, serotonergic synapse	0	0	1	0	0	0	High
PLCB1	mGluR, dopamine synapse, serotonergic synapse	0	0	0	1	0	0	High
PRKCA	mGluR, dopamine synapse, serotonergic synapse	0	0	0	1	0	0	High
AKT1	Dopamine synapse, cAMP	0	1	0	0	0	1	High
CACNA1B	Dopamine synapse, serotonergic synapse	0	0	0	0	0	1	High
CALM1	Dopamine synapse, cAMP	0	0	0	1	0	0	High
CAMK2A	Dopamine synapse, cAMP	0	0	0	0	0	1	High
CAMK2B	Dopamine synapse, cAMP	0	0	0	0	0	1	High
DRD3	Dopamine synapse, neuroactive ligand–receptor interaction	0	1	0	0	0	1	Low
GRIK5	mGluR, neuroactive ligand–receptor interaction	0	0	0	0	0	1	High
MAPK8	Dopamine synapse, cAMP	0	0	0	0	0	1	High
PLA2G4A	mGluR, serotonergic synapse	0	1	0	0	0	0	Low
PPP3CC	mGluR, dopamine synapse	0	1	0	0	0	0	High
PTGER3	Neuroactive ligand–receptor interaction, cAMP	0	0	0	0	0	1	Low
SLC18A1	Dopamine synapse, serotonergic synapse	0	1	0	0	0	0	Low
VIPR2	Neuroactive ligand–receptor interaction, cAMP	0	0	1	0	0	0	Low
**Hub genes from gene set B**								
CAMK2G	mGluR, dopamine synapse, cAMP, neuroactive ligand–receptor interaction	0	0	0	1	1	0	High
MYC	mGluR, dopamine synapse, cAMP, serotonergic synapse	0	0	0	1	0	0	High
SDCBP	mGluR, dopamine synapse, cAMP, neuroactive ligand–receptor interaction	0	0	0	0	0	1	High
ARNT	Dopamine synapse, cAMP, serotonergic synapse	0	1	0	0	0	0	High
EPB41L1	Dopamine synapse, neuroactive ligand–receptor interaction, cAMP	0	0	0	0	0	1	High
ERBB2	Dopamine synapse, neuroactive ligand–receptor interaction, cAMP	0	0	0	0	0	1	High
FSCN1	mGluR, dopamine synapse, serotonergic synapse	0	0	0	1	1	1	High
SLC9A3R1	mGluR, dopamine synapse, serotonergic synapse	0	0	0	0	0	1	High
AURKA	Dopamine synapse, cAMP	0	0	0	0	0	1	Low
CDK4	Dopamine synapse, cAMP	0	0	0	1	0	1	High
CLIC6	Dopamine synapse, neuroactive ligand–receptor interaction	0	0	0	0	0	1	Low
CMTM4	Neuroactive ligand–receptor interaction, cAMP	0	0	0	1	0	0	High
DCTN1	Dopamine synapse, cAMP	0	0	0	0	0	1	High
DERL1	cAMP, serotonergic synapse	0	0	0	1	1	0	High
DGUOK	Dopamine synapse, cAMP	0	0	0	0	0	1	High
EIF2AK3	Dopamine synapse, cAMP	0	0	0	1	0	0	High
FUS	Dopamine synapse, cAMP	0	0	0	1	0	0	High
GNB2	Dopamine synapse, cAMP	0	0	0	1	0	1	High
GRID2	mGluR, neuroactive ligand–receptor interaction	0	0	0	0	0	1	Low
HIST1H1C	Dopamine synapse, serotonergic synapse	0	0	0	1	0	0	High
ILK	Dopamine synapse, cAMP	0	0	0	0	0	1	High
ITPR1	Dopamine synapse, cAMP	0	0	0	1	0	1	High
MAP2K1	Dopamine synapse, cAMP	0	0	0	0	0	1	High
PPP1CB	Dopamine synapse, cAMP	0	0	0	0	0	1	High
RAF1	Dopamine synapse, cAMP	0	0	0	1	0	0	High
SLC39A1	Neuroactive ligand–receptor interaction, cAMP	0	0	0	1	0	0	High
SNCG	cAMP, serotonergic synapse	0	0	0	0	0	1	High
UBR5	Dopamine synapse, cAMP	0	0	0	1	0	1	High
XPO1	Dopamine synapse, cAMP	0	0	0	1	1	1	High
YWHAQ	Dopamine synapse, cAMP	0	0	0	0	0	1	High

## Data Availability Statement

The original contributions presented in the study are included in the article/Supplementary Material, further inquiries can be directed to the corresponding authors.

## Ethics Statement

All methods were carried out in accordance with relevant guidelines and regulations, and all experimental protocols were approved by the Children's Hospital of Philadelphia (CHOP) Institutional Review Board (IRB). Written informed consent to participate in this study was provided by the participants' legal guardian/next of kin.

## Author Contributions

HH and YL contributed to conceptualization. YL and H-QQ performed the literature search and took charge of the original draft writing. YL, H-QQ, XC, LT, and JG contributed to data preparation and analysis. YL, H-QQ, and HH contributed to review and revision. HH took charge of supervision. All authors contributed to data interpretation.

## Funding

The study was supported by the Institutional Development Funds from the Children's Hospital of Philadelphia to the Center for Applied Genomics. The Children's Hospital of Philadelphia Endowed Chair in Genomic Research to HH.

## Conflict of Interest

The authors declare that the research was conducted in the absence of any commercial or financial relationships that could be construed as a potential conflict of interest.

## Publisher's Note

All claims expressed in this article are solely those of the authors and do not necessarily represent those of their affiliated organizations, or those of the publisher, the editors and the reviewers. Any product that may be evaluated in this article, or claim that may be made by its manufacturer, is not guaranteed or endorsed by the publisher.
