# Supplementary material for: Expansion of Schizophrenia Gene Network Knowledge Using Machine Learning Selected Signals From Dorsolateral Prefrontal Cortex and Amygdala RNA-seq Data
Source: Front Psychiatry. 2022 Mar 21;13:797329. doi: 10.3389/fpsyt.2022.797329 (PMC8978801; doi:10.3389/fpsyt.2022.797329)
Supplement: Supplementary file 1 [file Table_1.DOCX]

MSSM_RNA_PFC_1

MSSM_RNA_PFC_100

MSSM_RNA_PFC_101

MSSM_RNA_PFC_114

MSSM_RNA_PFC_12

MSSM_RNA_PFC_13

MSSM_RNA_PFC_132

MSSM_RNA_PFC_183

MSSM_RNA_PFC_197

MSSM_RNA_PFC_201

MSSM_RNA_PFC_250

MSSM_RNA_PFC_265

MSSM_RNA_PFC_266

MSSM_RNA_PFC_336

MSSM_RNA_PFC_341

MSSM_RNA_PFC_343

MSSM_RNA_PFC_347

MSSM_RNA_PFC_348

MSSM_RNA_PFC_350

MSSM_RNA_PFC_351

MSSM_RNA_PFC_352

MSSM_RNA_PFC_357

MSSM_RNA_PFC_44

MSSM_RNA_PFC_59

MSSM_RNA_PFC_96

PENN_RNA_PFC_33

PENN_RNA_PFC_34

PENN_RNA_PFC_35

PENN_RNA_PFC_38

PENN_RNA_PFC_39

PENN_RNA_PFC_40

PENN_RNA_PFC_41

PENN_RNA_PFC_42

PENN_RNA_PFC_43

PENN_RNA_PFC_44

PENN_RNA_PFC_45

PENN_RNA_PFC_46

PENN_RNA_PFC_48

PENN_RNA_PFC_49

PENN_RNA_PFC_50

PENN_RNA_PFC_51

PENN_RNA_PFC_52

PENN_RNA_PFC_54

PENN_RNA_PFC_55

PENN_RNA_PFC_56

PENN_RNA_PFC_58

PENN_RNA_PFC_59

PENN_RNA_PFC_61

PENN_RNA_PFC_62

PENN_RNA_PFC_63

PENN_RNA_PFC_64

PENN_RNA_PFC_65

PENN_RNA_PFC_66

PENN_RNA_PFC_67

PENN_RNA_PFC_68

PENN_RNA_PFC_69

PENN_RNA_PFC_70

PENN_RNA_PFC_71

PENN_RNA_PFC_73

PENN_RNA_PFC_74

PENN_RNA_PFC_76

PENN_RNA_PFC_77

PENN_RNA_PFC_78

PENN_RNA_PFC_79

PENN_RNA_PFC_80

PENN_RNA_PFC_81

PENN_RNA_PFC_88

PENN_RNA_PFC_96

PENN_RNA_PFC_97

PENN_RNA_PFC_98

PENN_RNA_PFC_99

PENN_RNA_PFC_100

PENN_RNA_PFC_101

PENN_RNA_PFC_103

PENN_RNA_PFC_104

PENN_RNA_PFC_105

PENN_RNA_PFC_106

PENN_RNA_PFC_107

PENN_RNA_PFC_108

PENN_RNA_PFC_109

PITT_RNA_PFC_10020

PITT_RNA_PFC_10026

PITT_RNA_PFC_1088

PITT_RNA_PFC_1105

PITT_RNA_PFC_1173

PITT_RNA_PFC_1188

PITT_RNA_PFC_1189

PITT_RNA_PFC_1209

PITT_RNA_PFC_1211

PITT_RNA_PFC_1222

PITT_RNA_PFC_1230

PITT_RNA_PFC_1256

PITT_RNA_PFC_1263

PITT_RNA_PFC_1341

PITT_RNA_PFC_1361

PITT_RNA_PFC_1367

PITT_RNA_PFC_1420

PITT_RNA_PFC_1453

PITT_RNA_PFC_1454

PITT_RNA_PFC_1455

PITT_RNA_PFC_1474

PITT_RNA_PFC_1506

PITT_RNA_PFC_1542

PITT_RNA_PFC_1579

PITT_RNA_PFC_1581

PITT_RNA_PFC_1691

PITT_RNA_PFC_1712

PITT_RNA_PFC_1734

PITT_RNA_PFC_533

PITT_RNA_PFC_539

PITT_RNA_PFC_566

PITT_RNA_PFC_581

PITT_RNA_PFC_621

PITT_RNA_PFC_622

PITT_RNA_PFC_640

PITT_RNA_PFC_802

PITT_RNA_PFC_829

PITT_RNA_PFC_904

PITT_RNA_PFC_917

PITT_RNA_PFC_933

MSSM_RNA_PFC_102

MSSM_RNA_PFC_108

MSSM_RNA_PFC_109

MSSM_RNA_PFC_111

MSSM_RNA_PFC_112

MSSM_RNA_PFC_113

MSSM_RNA_PFC_116

MSSM_RNA_PFC_117

MSSM_RNA_PFC_118

MSSM_RNA_PFC_119

MSSM_RNA_PFC_120

MSSM_RNA_PFC_123

MSSM_RNA_PFC_124

MSSM_RNA_PFC_125

MSSM_RNA_PFC_126

MSSM_RNA_PFC_127

MSSM_RNA_PFC_131

MSSM_RNA_PFC_133

MSSM_RNA_PFC_134

MSSM_RNA_PFC_135

MSSM_RNA_PFC_136

MSSM_RNA_PFC_141

MSSM_RNA_PFC_142

MSSM_RNA_PFC_143

MSSM_RNA_PFC_15

MSSM_RNA_PFC_153

MSSM_RNA_PFC_157

MSSM_RNA_PFC_158

MSSM_RNA_PFC_16

MSSM_RNA_PFC_163

MSSM_RNA_PFC_175

MSSM_RNA_PFC_176

MSSM_RNA_PFC_2

MSSM_RNA_PFC_210

MSSM_RNA_PFC_241

MSSM_RNA_PFC_297

MSSM_RNA_PFC_3

MSSM_RNA_PFC_300

MSSM_RNA_PFC_302

MSSM_RNA_PFC_46

PENN_RNA_PFC_1

PENN_RNA_PFC_3

PENN_RNA_PFC_4

PENN_RNA_PFC_5

PENN_RNA_PFC_6

PENN_RNA_PFC_7

PENN_RNA_PFC_9

PENN_RNA_PFC_11

PENN_RNA_PFC_14

PENN_RNA_PFC_17

PENN_RNA_PFC_18

PENN_RNA_PFC_19

PENN_RNA_PFC_20

PENN_RNA_PFC_21

PENN_RNA_PFC_23

PENN_RNA_PFC_24

PENN_RNA_PFC_27

PENN_RNA_PFC_28

PENN_RNA_PFC_31

PENN_RNA_PFC_32

PENN_RNA_PFC_83

PENN_RNA_PFC_86

PENN_RNA_PFC_87

PENN_RNA_PFC_91

PENN_RNA_PFC_92

PENN_RNA_PFC_95

PITT_RNA_PFC_10003

PITT_RNA_PFC_10005

PITT_RNA_PFC_1026

PITT_RNA_PFC_1047

PITT_RNA_PFC_1086

PITT_RNA_PFC_1099

PITT_RNA_PFC_1122

PITT_RNA_PFC_1201

PITT_RNA_PFC_1247

PITT_RNA_PFC_1336

PITT_RNA_PFC_1350

PITT_RNA_PFC_1372

PITT_RNA_PFC_1374

PITT_RNA_PFC_1386

PITT_RNA_PFC_1391

PITT_RNA_PFC_1433

PITT_RNA_PFC_1472

PITT_RNA_PFC_1518

PITT_RNA_PFC_1524

PITT_RNA_PFC_1543

PITT_RNA_PFC_1554

PITT_RNA_PFC_1555

PITT_RNA_PFC_1558

PITT_RNA_PFC_1583

PITT_RNA_PFC_1635

PITT_RNA_PFC_1792

PITT_RNA_PFC_546

PITT_RNA_PFC_551

PITT_RNA_PFC_604

PITT_RNA_PFC_630

PITT_RNA_PFC_634

PITT_RNA_PFC_681

PITT_RNA_PFC_685

PITT_RNA_PFC_686

PITT_RNA_PFC_694

PITT_RNA_PFC_700

PITT_RNA_PFC_739

PITT_RNA_PFC_806

PITT_RNA_PFC_818

PITT_RNA_PFC_852

PITT_RNA_PFC_857

PITT_RNA_PFC_902

PITT_RNA_PFC_970

PITT_RNA_PFC_987

PITT_RNA_PFC_988

PITT_RNA_BP_PFC_1031

PITT_RNA_BP_PFC_1081

PITT_RNA_BP_PFC_1153

PITT_RNA_BP_PFC_1196

PITT_RNA_BP_PFC_1293

PITT_RNA_BP_PFC_1298

PITT_RNA_BP_PFC_1394

PITT_RNA_BP_PFC_1403

PITT_RNA_BP_PFC_1444

PITT_RNA_BP_PFC_1482

PITT_RNA_BP_PFC_1489

PITT_RNA_BP_PFC_1598

PITT_RNA_BP_PFC_1605

PITT_RNA_BP_PFC_1637

PITT_RNA_BP_PFC_1694

PITT_RNA_BP_PFC_1770

PITT_RNA_BP_PFC_1783

PITT_RNA_BP_PFC_1789

PITT_RNA_BP_PFC_789

PITT_RNA_BP_PFC_795

PITT_RNA_BP_PFC_838

PITT_RNA_BP_PFC_840

PITT_RNA_BP_PFC_841
